# A. F. A. King, M. D.

**Published:** 1890-06

**Authors:** A. F. A. King

**Affiliations:** Washington, D. C.


WASHINGTON, D. C., May 1, 1890.

*Editors Buffalo Medical and Surgical Journal:*

WILL you kindly allow space in your columns for the following communication?

You were good enough, some months ago, to send me marked copies of your valuable JOURNAL—the number for January, 1890—on pages 382 and 383 of which you comment on the annual announcement of the Medical Department of the University of Vermont containing a circular advertising "Lactated Food" and "Celery Compound." You say: "We suppose this indicates some of the therapeutical measures used in this institution." You further state: "This is the first time, to our knowledge, that the annual announcement of a reputable medical school was ever used as an advertising medium for proprietary medicines;" and, "when medical colleges become the advertising agents of," etc., "they lower the standard of their usefulness and affront the dignity of the profession." In this latter statement I must say I agree with you. However, what I most desire in this note is to convey to you my sympathy for that dreadful lack of knowledge which leads you to suppose that the University of the Green Mountain State is the only transgressor. I have not seen the "Lactated Food" circular to which you refer, but I am informed it was a loose leaf, or sheet, slipped into the college circular, after printing, and not published by the college authorities. But that is not the point; it is undignified either way, and needs reform.

But reform, good Messrs. Editors, like charity, should begin at home. Why do you assail the diminutive "mote" in the eye of your neighbor Vermont, when a huge "beam," more than twenty times as offensive, is flauntingly displayed in your own great "Empire" State of New York?

Herewith I send you the eighth announcement of the "New York Post-Graduate School and Hospital"—sessions of 1889–'90. It contains twenty-two pages of advertisements, not slipped into, but bound with the college circular. Among them you will find: Fehr's "Compound Talcum Baby Powder;" "Imperial Granum;" "Syrupus Roborans;" "Planten's Capsules;" "Exalgine;" "Liquid Albolene;" "Hollow Suppositories," and other proprietary medicines, not to mention "steel pens," "saddle-bags," "photographic instruments," and last, but not least, cigarettes"—"Richmond Straight

Cut" and "High-Grade Cigarettes"—the former being illustrated with the smiling face and broad-brimmed hat of a now familiar figure, puffing smoke; while the "High-Grade" brand is magnificently embellished with the picture of a female modestly pulling up her clothes to a *high grade* above her ankle, in the presence of a male, whose cigarette she seems to be lighting from her own. Can you suppose, Messrs. Editors, that these things "indicate some of the therapeutical measures used in this institution"—the Post-graduate Medical School—in the same way that you supposed the "Lactated Food" and "Celery Compound" circulars indicated the use of these preparations by the University of Vermont?

This is all. I shall be glad to hear or read any future statement you may be disposed to make in reply, now that you have a wider knowledge of facts.

The University of Vermont is an institution with which I have been associated for nearly twenty years. Its methods of teaching, and requirements for graduation, etc., reflect credit upon the profession. It has succeeded in growing into a large school, and has, therefore, invited opposition and, perhaps, some jealousy. I cannot be silent when it has been unjustly assailed. R. S. V. P.

A. F. A. KING, M. D.

---

DR. L. S. PILCHER, of Brooklyn, N. Y., recently read a paper on Cranial and Spinal Injuries (*Annals of Surgery*, March, 1890), in which he stated that the results in these cases are calculated to prevent the indulgence of too sanguine expectations in approaching similar cases. Nevertheless, although, in the great majority of cases, either the extent or the seat of the lesion will render all attempts at surgical relief futile, yet in some cases, intracranial conditions susceptible of relief and cure do exist, and in any case in which a reasonable doubt exists, it seemed to him to be justifiable to give the benefit of the doubt to the patient, especially when it is evident that without intervention a fatal termination is certainly imminent.—*College and Clinical Record.*

AT a recent meeting of the Board of Health of Philadelphia, Dr. Keyser offered a resolution, which was adopted without debate, that the medical inspector be instructed to inquire what disposition is made of coffins after bodies have been cremated, especially in the event of being used for bodies of persons who have died of contagious diseases. It is said that coffins have been carried off by undertakers to be used a second time.—*Boston Medical and Surgical Journal.*

